# The Predictive Value of Different Nutritional Indices Combined with the GRACE Score in Predicting the Risk of Long-Term Death in Patients with Acute Coronary Syndrome Undergoing Percutaneous Coronary Intervention

**DOI:** 10.3390/jcdd9100358

**Published:** 2022-10-17

**Authors:** Xu Chen, Shiqiang Xiong, Yingzhong Chen, Lianchao Cheng, Qiang Chen, Siqi Yang, Lingyao Qi, Hanxiong Liu, Lin Cai

**Affiliations:** Department of Cardiology, The Third People’s Hospital of Chengdu, Affiliated Hospital of Southwest Jiaotong University, Chengdu 610014, China

**Keywords:** geriatric nutritional risk index, prognostic nutritional index, GRACE score, acute coronary syndrome, percutaneous coronary intervention, long-term prognosis, all-cause death, nutritional status

## Abstract

Nutritional status is associated with prognosis in acute coronary syndrome (ACS) patients. Although the Global Registry of Acute Coronary Events (GRACE) risk score is regarded as a relevant risk predictor for the prognosis of ACS patients, nutritional variables are not included in the GRACE score. This study aimed to compare the prognostic ability of the Geriatric Nutritional Risk Index (GNRI) and Prognostic Nutritional Index (PNI) in predicting long-term all-cause death in ACS patients undergoing percutaneous coronary intervention (PCI) and to determine whether the GNRI or PNI could improve the predictive value of the GRACE score. A total of 799 patients with ACS who underwent PCI from May 2018 to December 2019 were included and regularly followed up. The performance of the PNI in predicting all-cause death was better than that of the GNRI [C-index, 0.677 vs. 0.638, *p* = 0.038]. The addition of the PNI significantly improved the predictive value of the GRACE score for all-cause death [increase in C-index from 0.722 to 0.740; IDI 0.006; NRI 0.095; *p* < 0.05]. The PNI was superior to the GNRI in predicting long-term all-cause death in ACS patients undergoing PCI. The addition of the PNI to the GRACE score could significantly improve the prediction of long-term all-cause death.

## 1. Introduction

Coronary atherosclerotic heart disease (CAD) is a significant health concern [1], and acute coronary syndrome (ACS) is associated with a high prevalence of morbidity and mortality worldwide [2]. In recent years, with the development of reperfusion therapy and the improvement of comprehensive cardiac rehabilitation in patients with ACS, the prognosis of ACS patients has improved [3,4]; however, the long-term prognosis of ACS patients is still poor [2]. Therefore, accurate risk prediction in ACS patients is critically important.

The Global Registry of Acute Coronary Events (GRACE) risk score for mortality and reinfarction up to 6 months post-discharge can be used to estimate the mortality risk 6 months after discharge in patients with ACS and has been shown to retain good discrimination of mortality up to 4 years in ACS patients [5,6,7]. The GRACE score is recommended in current guidelines for risk stratification and prognostic evaluation in ACS patients [8]. Although recent studies have demonstrated that nutritional status is associated with prognosis in ACS patients [9,10], nutritional variables are not included in the GRACE score model for risk prediction.

Nutritional indices have been primarily designed to evaluate malnutrition-related risk. Patients with ACS in a poor nutritional state might have a worse prognosis [9,10]. Recent studies have shown that a low Geriatric Nutritional Risk Index (GNRI) [11] is an independent predictor of poor prognosis in CAD patients after percutaneous coronary intervention (PCI) [12,13], and a low level of the Prognostic Nutritional Index (PNI) [14] is independently associated with a higher risk of all-cause death in ACS patients after PCI [15,16]. At present, few studies have focused on whether the addition of a nutritional index improves the predictive ability of the GRACE score in patients with ACS undergoing PCI [9].

Therefore, this study aimed to compare the prognostic prediction value of the GNRI and PNI in predicting long-term all-cause mortality in patients with ACS after PCI and explore whether the GNRI or PNI could improve the predictive value of GRACE score-based prognostic models for predicting long-term all-cause death in ACS patients.

## 2. Materials and Methods

### 2.1. Study Population

This study was a single-centre, bidirectional, observational cohort study. Patients who were diagnosed with ACS undergoing PCI at the Third People’s Hospital of Chengdu from May 2018 to December 2019 were included. ACS was defined according to the guidelines issued by the Chinese Society of Cardiology for the diagnosis of patients with ST-elevation myocardial infarction, non-ST elevation myocardial infarction, or unstable angina [17,18]. The exclusion criteria were as follows: (1) missing important clinical trial data; (2) PCI failure or in-hospital death; (3) severe acute infection or chronic infection; (4) severe renal insufficiency or liver dysfunction; (5) malignant tumour; (6) other severe cardiac conditions, namely, severe valvular heart disease, constrictive pericarditis, myocarditis, nonischaemic cardiomyopathy, congenital heart disease, rheumatic heart disease or other serious heart diseases; coronary vasculitis, systemic sclerosis, systemic vasculitis, systemic lupus erythematosus or other connective tissue diseases, to avoid other diseases affecting the long-term prognosis; (7) blood system diseases, immune system diseases, and recent use of immunosuppressive medication to avoid affecting the lymphocyte count; and (8) loss to follow-up.

### 2.2. Data Collection

Clinical baseline data of enrolled patients were collected through the electronic medical record system. The collected data included demographic data, personal history and past medical history, diagnoses, symptoms and clinical signs at admission, laboratory test and auxiliary examination results, and records of PCI surgery and medication at hospital discharge.

### 2.3. Calculation of Nutritional Indices and GRACE Scores

The GNRI [11], which is based on serum albumin level, body weight, and height, assesses the nutritional status of patients with various pathological conditions. The calculation formula is as follows [11]: 1.489 × serum albumin (g/L) + 41.7 × (measured body weight (kg)/ideal body weight (kg)). The ideal body weight was calculated as follows: body height (cm) − 100 − {[body height (cm) − 150]/4} for males, and body height (cm) – 100 − {[body height (cm) − 150]/2.5} for females. When the measured body weight was higher than the ideal weight, the measured body weight/ideal body weight was set to 1. According to the baseline GNRI, patients were classified into 4 malnutrition risk categories: no nutritional risk (GNRI > 98); mild malnutrition risk (GNRI 92~98); moderate malnutrition risk (GNRI 82~91) and severe malnutrition risk (GNRI < 82). Furthermore, weight loss was defined as a measured body weight lower than the ideal weight. To estimate nutritional status, the PNI [14] is based on a combination of serum albumin level and total lymphocyte count. The calculation formula is as follows [14]: 1 × serum albumin (g/L) + 5 × total lymphocyte count (×10^9^/L). According to the baseline PNI, patients were classified into 3 malnutrition risk categories [19]: no nutritional risk (PNI > 38), moderate malnutrition risk (PNI 35~38) and severe malnutrition risk (PNI < 35). The web-based GRACE score calculator was used to calculate the risk of death from discharge to 6 months for each patient [5].

### 2.4. Follow-Up for All-Cause Death

Clinical follow-up was scheduled for 1, 6, and 12 months after discharge and once a year thereafter, and follow-up data were obtained from a telephone questionnaire and/or electronic medical records. Follow-up was performed to observe the occurrence of all-cause death and to record the time of all-cause death.

### 2.5. Statistical Analyses

Statistical analysis was performed with IBM SPSS Statistics version 26.0 (IBM Corporation, Chicago, IL, USA) and R statistical programming language version 4.1.2 (Vienna, Austria). Categorical variables were expressed as percentages, and the chi-square test or Fisher’s exact probability method was used for comparisons between groups. The continuous variates were first tested for normality. Data with a normal distribution were expressed as the mean and standard deviation, and comparisons between groups were conducted with the independent sample *t* test; data with a nonnormal distribution were expressed as the median and quartiles [M (IQR)], and comparisons between groups were performed with the Mann-Whitney U test. The cumulative incidence of all-cause death was compared across groups using Kaplan-Meier survival curves and a log-rank test. Univariate Cox regression analysis was performed to determine the association between baseline characteristics and all-cause death. Variables with a *p* value < 0.05 in a univariate Cox regression analysis were included in the multivariate analysis. In addition, PNI, GNRI and nutrition-related factors were added separately for multivariate Cox regression analysis to investigate whether these nutrition-related factors were independent predictors of long-term all-cause mortality in ACS patients.

The concordance index (C-index) of the GNRI and PNI in predicting long-term all-cause death was calculated and compared. To evaluate the ability of these nutritional indices to improve the predictive value of the GRACE risk model, these indices were added to the GRACE risk score as new models, and C-index, net reclassification improvement (NRI) and integrated discrimination improvement (IDI) statistical analyses were performed. A *p* value (two-tailed) < 0.05 was considered significant.

## 3. Results

### 3.1. Baseline Characteristics

This study included 799 patients; 72.3% were males, and the mean age was 66.26 ± 11.34 years. During the median follow-up of 30 months (IQR: 25–35 months), all-cause death was noted for 46 patients (5.8%). Patients were divided into two groups: the all-cause death group (46 patients who died during follow-up) and the survival group (753 patients). Clinical baseline characteristics between the two groups are shown in Table 1. The PNI (43.86 ± 5.35 vs. 47.76 ± 5.74, *p* < 0.001) and GNRI [96.94 (92.33, 101.71) vs. 100.37 (95.23, 104.09), *p* = 0.002] were significantly lower in the all-cause death group than in the survival group, and the GRACE scores were higher [134 (114, 153) vs. 104 (85, 129)], *p* < 0.001]. Among the nutrition-related indicators used in the nutritional indices, the serum albumin level and total lymphocyte count in the all-cause death group were significantly lower (*p* < 0.05 for all); however, there was no significant difference in the proportion of weight loss between the two groups. Compared to patients in the survival group, those in the all-cause death group were older and more likely to have a previous history of CAD, atrial fibrillation, diabetes, and congestive heart failure (*p* < 0.05 for all); the proportion of smokers in the all-cause death group was lower (*p* < 0.05). In terms of the laboratory examinations, a higher proportion of patients in the all-cause death group had elevated cardiac enzymes (*p* < 0.05). Triglyceride, haemoglobin, haematocrit, and left ventricular ejection fraction levels were all lower in the all-cause death group (*p* < 0.05). Finally, lower proportions of patients in the all-cause death group were discharged and received dual antiplatelet drug treatment (*p* < 0.05).

### 3.2. Cumulative Incidence of All-Cause Death in Patients in Different Nutritional Risk Groups

**Figure 1:**
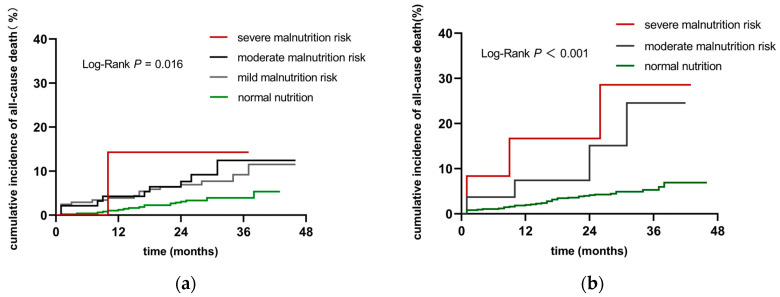
Comparison of cumulative incidence of all-cause death during follow-up (**a**) Cumulative incidence of all-cause death stratified by the GNRI; (**b**) Cumulative incidence of all-cause death stratified by the PNI.

As shown in Figure 1, according to the GNRI score, the patients were divided into a normal nutrition group, *n* = 493; a mild malnutrition risk group, *n* = 205; a moderate malnutrition risk group, *n* = 94; and a severe malnutrition risk group, *n* = 7. The Kaplan-Meier analysis indicated that patients in the mild to severe malnutrition risk groups had a significantly higher cumulative incidence of all-cause death than those in the normal nutrition group (log-rank *p* = 0.016). According to the PNI score, the patients were divided into a normal nutrition group, *n* = 760; a moderate malnutrition risk group, *n* = 27; and a severe malnutrition risk group, *n* = 12. The Kaplan-Meier curve showed that patients with moderate or severe malnutrition risk had a significantly worse prognosis than patients without malnutrition risk (log-rank *p* < 0.001).

### 3.3. The Relationship between Different Nutritional Indices and All-Cause Mortality

The multivariate Cox regression analysis showed that the GRACE score [HR 1.017, 95% CI (1.006–1.029), *p* = 0.002] and atrial fibrillation [HR 2.249, 95% CI (1.007–5.024), *p* = 0.048] were independent predictors for all-cause death (Table 2).

Serum albumin, total lymphocyte count, weight loss, the PNI and the GNRI were added into the multivariate Cox regression model above separately. The results are shown in Table 3. Serum albumin [HR 0.918, 95% CI (0.844–0.998), *p* = 0.044] and the PNI [HR 0.926, 95% CI (0.867–0.989), *p* = 0.022] were independent predictors for long-term all-cause death in ACS patients who underwent PCI, total lymphocyte count, weight loss and GNRI may not be independent predictors for all-cause death.

The performance of the PNI in predicting long-term all-cause death [C-index 0.677, 95% CI (0.603–0.752)] was better than that of the GNRI [C-index 0.638, 95% CI (0.560–0.715)], and the difference was statistically significant (*p* < 0.001).

### 3.4. Additional Predictive Values of the Nutritional Indices in the GRACE Risk Prediction Model

The addition of the PNI to the GRACE score significantly improved the prediction of all-cause death in patients with ACS, increasing the C-index from 0.722 to 0.740 (*p* = 0.027); the NRI was 0.095 (95% CI, 0.004–0.147, *p* < 0.001), and the IDI was 0.006 (95% CI, 0.000–0.014, *p* < 0.001). The addition of the GNRI also improved the predictive performance of long-term all-cause death, but the optimization effect was not significant (Table 4).

## 4. Discussion

The results of this study demonstrated that the PNI was an independent predictor of long-term all-cause death after PCI in ACS patients, while the GNRI was not. The PNI was superior to the GNRI in predicting long-term all-cause death; therefore, it is effective and feasible to use the PNI for nutritional risk assessment in ACS patients undergoing PCI. The addition of the PNI to the GRACE score could significantly improve the predictive value of these assessments for long-term all-cause death and optimize the prognosis prediction in ACS patients undergoing PCI.

Numerous indices have been used in previous studies to assess the nutritional status of patients, such as the GNRI, PNI, Triglycerides × Total Cholesterol × Body Weight Index (TCBI) [20] and Controlling Nutritional Status (CONUT) score [21]. The CONUT and TCBI are based on total cholesterol or triglyceride levels to evaluate the nutritional status, and high total cholesterol and triglyceride levels indicate good nutritional status. However, total cholesterol and triglyceride levels are important risk factors for the onset and prognosis of ACS patients [22,23]. And the assessment of nutritional status by these two indices might be influenced by the use of statins. Therefore, in this study, the GNRI and PNI were applied to assess the nutritional status of patients with ACS.

### 4.1. Predictive Value of Serum Albumin for All-Cause Death

Both the PNI and GNRI use serum albumin as a nutritional risk assessment indicator. Serum albumin is the most abundant protein in human blood plasma, and several factors may influence the serum albumin concentration; among these factors, malnutrition and inflammation may be the main cause of reduced serum albumin levels [24,25]. Low serum albumin levels have been identified as a risk factor for the development of coronary artery disease [26,27]; moreover, in patients with ACS, lower serum albumin levels tend to predict worse outcomes [28]. Serum albumin has antioxidant and anti-inflammatory activities and inhibits platelet aggregation, and anticoagulation activation [29], so lower serum albumin levels contribute to accelerated atherosclerosis in several ways. In addition, as a chronic inflammatory disease, atherosclerosis correlates with increased production of catabolic cytokines, muscle catabolism, and appetite suppression, thus causing a decline in albumin levels [30]. This may result in a vicious cycle that promotes atherosclerosis and decreases serum albumin. In our study, the all-cause death group had significantly lower serum albumin levels than the survival group, and serum albumin was an independent predictor for all-cause death in ACS patients who underwent PCI, which was consistent with previous findings [28,31]. Therefore, it is reasonable to use serum albumin as one of the indicators for predicting the prognosis of patients with ACS.

### 4.2. Predictive Value of Weight Loss for All-Cause Death

Consistent with a retrospective cohort study involving 5062 ACS patients in 2020 [9], we also found that the predictive value of the GNRI for all-cause death was inferior to that of the PNI, which may be reflected in the difference in the predictive value of weight loss and total lymphocyte count for all-cause death.

To avoid failing to identify the nutritional status of obese patients, the GNRI formula requires that the ratio of the patient’s measured body weight to the ideal body weight be calculated based on the patient’s height and weight, and all ratios greater than 1 are defined as 1. However, the measured body weight of 623 patients in this study was higher than their ideal weight, accounting for 78.0% of the participants, and the difference in nutritional risk was only reflected in the serum albumin level in this group of patients; therefore, the predictive value of the GNRI for all-cause death may be inferior to that of the PNI. Another reason may be that the proportion of weight loss did not differ between the all-cause death group and the survival group; these results differ somewhat from previous studies [32,33]. Previous studies have reported that weight loss is significantly associated with a poor prognosis in patients with CAD [32]. Based on previous studies, weight loss was defined as BMI < 18.5 or BMI < 20 [32,33]. The GNRI definition of weight loss as measured weight less than ideal weight is different from the BMI definition of weight loss, which may be one of the reasons why weight loss is a poor predictor of patient prognostic risk in this study. Another reason why weight loss was not associated with all-cause death in this study may be that the patients in the all-cause death group had lower serum albumin levels and were more likely to have renal dysfunction; these reasons could lead to sodium and water retention and weight gain, so body weight in these patients may not reflect their true nutritional status.

### 4.3. Predictive Value of Total Lymphocyte Counts for All-Cause Death

The PNI added the total number of lymphocytes to serum albumin to assess nutritional risk, which has good predictive value for all-cause death of ACS patients after PCI. Lymphocytes are important immune cells involved in the processes of atherosclerosis [34]. Lymphocyte subpopulations have different impacts on the development of atherosclerosis. Stimulated CD4+ T lymphocytes can differentiate into effector T-cell (Teff) or regulatory T-cell (Treg) subsets; Teff responses promote atherosclerotic disease, while Tregs have been shown to induce the regression of atherosclerosis and increase plaque stability [35]. In addition, CD8+ T cells exert cytotoxic functions in atherosclerotic plaques and promote macrophage cell death and necrotic core formation, whereas subsets of regulatory CD25+CD8+ T cells with immunosuppressive functions can inhibit the development of atherosclerosis [36]. Although B lymphocytes are relatively small in number compared to T lymphocytes and are also thought to be an important regulator of pro- and anti-inflammatory effects in atherosclerosis, B1 lymphocytes appear to attenuate atherosclerosis, whereas B2 lymphocytes can aggravate this process [37].

Compared with healthy people, patients with ACS have significantly lower total lymphocyte counts [38,39]. In cases of stress, the release of cortisol and catecholamines in the blood increases, which leads to bone marrow suppression; thus, the proliferation and differentiation of lymphocytes are downregulated, and the apoptosis of lymphocytes is aggravated [40,41]. Previous studies have shown that the total lymphocyte count of CAD patients with major adverse cardiovascular events (MACEs; all-cause death; non-fatal myocardial infarction; non-fatal stroke; clinical-driven target vessel revascularization) after PCI is significantly lower than that of patients without MACEs [40,42]. We found that the total lymphocyte counts of patients in the all-cause death group were lower than those in the survival group, which was consistent with previous studies.

### 4.4. Predictive Value of the PNI Combined with the GRACE Score for All-Cause Death

The GRACE score, which includes several important prognostic factors for patients with ACS, has been proven to be an effective predictive model for the prognosis of ACS patients [5]. With the deepening of clinical studies, nutritional status has been found to be an important predictor of prognosis in patients with ACS; however, it is not included in the GRACE score for risk estimation. Nutritional status can be assessed in a variety of ways. The nutritional index can be used to assess the nutritional status of patients with simple information, such as common laboratory indicators and height and weight, and is not only effective but also suitable for the clinical fast-paced work environment. However, the nutritional index alone may not be as comprehensive as the GRACE score in predicting the prognosis of ACS patients. Our results indicate that the combination of the PNI with the GRACE score may potentially help with risk stratification and the administration of effective measures to improve the clinical outcomes of ACS patients.

Currently, there is no recommended method for the evaluation of the nutritional status of ACS patients [17,18,43]. This study provided clinical evidence for the nutritional status assessment of ACS patients after PCI. We demonstrate that the PNI is effective in stratifying the nutritional status of ACS patients in an East Asian population. The PNI is easy to perform as a preliminary screening of the nutritional status of patients and would be easy to promote for daily clinical practice. In addition, targeted nutritional guidance can be given to patients with high nutritional risk to optimize their nutritional status and improve their prognosis.

The limitations of this study include the following points. First, this study was a single-centre study, and the number of patients with all-cause death events was small, thus limiting the statistical significance. Second, the samples in this study were selected from West China, which has a relatively homogeneous population; therefore, the results may not be generalizable to other regions. Third, serum albumin levels and lymphocyte counts were measured at baseline, and the follow-up period was not monitored, so the dynamic changes in the PNI during follow-up that impact patient outcomes remain unclear. Finally, the effect of the PNI on the GRACE score in prognostic prediction needs to be further verified by a large sample and multicentre prospective studies.

## 5. Conclusions

The PNI was an independent predictor of long-term all-cause death in ACS patients undergoing PCI, and the PNI was superior to the GNRI in predicting long-term all-cause death. The addition of the PNI to the GRACE score could significantly improve the predictive value of long-term all-cause death, which could optimize prognostic risk prediction for ACS patients undergoing PCI and thus take effective measures to improve the clinical outcomes of ACS patients.

## Figures and Tables

**Table 1:** Comparison of baseline characteristics of patients in the all-cause death group and the survival group.

Variable	All Subjects (*n* = 799)	All-Cause Death (*n* = 46)	Survival (*n* = 753)	*p* Value
Male, *n* (%)	578 (72.3)	30 (65.2)	548 (72.8)	0.266
PNI	47.53 ± 5.79	43.86 ± 5.35	47.76 ± 5.74	<0.001
GNRI	100.22 (95.01, 103.94)	96.94 (92.33, 101.71)	100.37 (95.23, 104.09)	0.002
Serum albumin, g/L	39.60 (36.60, 42.10)	38.10 (34.60, 40.30)	39.60 (36.80, 42.20)	0.001
Total lymphocyte count, ×10^9^/L	1.55 (1.17, 1.98)	1.23 (0.89, 1.70)	1.56 (1.19, 2.00)	0.001
Weight loss, *n* (%)	176 (22.0)	9 (19.6)	167 (22.2)	0.678
BMI, kg/m^2^	24.24 (22.58, 26.67)	24.44 (23.10, 26.49)	24.24 (22.54, 26.67)	0.875
GRACE score	106 (85, 131)	134 (114, 153)	104 (85, 129)	<0.001
Age, years	66.26 ± 11.34	73.41 ± 10.50	65.82 ± 11.25	<0.001
HR, bpm	76.00 (67.00, 85.00)	75.50 (67.00, 88.50)	76.00 (67.00, 85.00)	0.752
SBP, mmHg	130.00 (119.00, 144.00)	132.00 (114.75, 149.25)	130.00 (119.50, 144.00)	0.998
Scr, umol/L	75.90 (64.10, 89.80)	77.60 (60.53, 110.48)	75.70 (64.30, 88.90)	0.301
Congestive heart failure, *n* (%)	274 (34.3)	27 (58.7)	247 (32.8)	<0.001
History of MI, *n* (%)	49 (6.1)	5 (10.9)	44 (5.8)	0194
ST-Segment Depression, *n* (%)	374 (46.8)	25 (54.3)	349 (46.3)	0.291
Elevated Cardiac Enzymes, *n* (%)	533 (66.7)	38 (82.6)	495 (65.7)	0.018
Smoking, *n* (%)	317 (39.7)	10 (21.7)	307 (40.8)	0.010
Previous CAD, *n* (%)	180 (22.6)	16 (34.8)	164 (21.8)	0.041
Previous PCI, *n* (%)	63 (7.9)	4 (8.7)	59 (7.8)	0.778
Atrial fibrillation, *n* (%)	51 (6.4)	10 (21.7)	41 (5.4)	<0.001
Hypertension, *n* (%)	505 (63.2)	33 (71.7)	472 (62.7)	0.216
Diabetes, *n* (%)	270 (33.8)	22 (47.8)	248 (32.9)	0.038
Previous Stroke, *n* (%)	24 (3.0)	1 (2.2)	23 (3.1)	1.000
Renal dysfunction, *n* (%)	50 (6.3)	6 (13.0)	44 (5.8)	0.060
Hs-TnT, pg/mL	42.05 (11.55, 916.10)	137.10 (27.72, 1780.50)	38.71 (11.28, 859.50)	0.025
Uric acid, μmol/L	366.50 (308.10, 439.10)	400.25 (324.65, 478.95)	365.35 (307.43, 435.78)	0.053
TG, mmol/L	1.45 (1.06, 2.19)	1.22 (0.97, 1.66)	1.47 (1.07, 2.24)	0.017
TC, mmol/L	4.34 (3.62, 5.20)	4.33 (3.49, 5.08)	4.34 (3.63, 5.21)	0.853
LDL-C, mmol/L	2.66 (2.15, 3.31)	2.68 (2.07, 3.38)	2.66 (2.16, 3.31)	0.843
HDL-C, mmol/L	1.10 (0.95, 1.30)	1.13 (0.97, 1.37)	1.10 (0.95, 1.29)	0.486
Blood glucose, mmol/L	5.82 (5.01, 7.38)	6.19 (5.20, 8.26)	5.79 (5.01, 7.37)	0.107
Haemoglobin, g/L	137.00 (124.00, 148.00)	128.50 (117.75, 140.25)	137.00 (124.00, 148.00)	0.002
Haematocrit, %	41.30 (37.80, 44.20)	39.00 (35.05, 42.65)	41.40 (38.00, 44.30)	0.002
LVEF, %	58 (51, 62)	50 (40, 58)	58 (52, 62)	<0.001
MVD, *n* (%)	468 (63.7)	32 (76.2)	436 (62.9)	0.082
LM or LAD, *n* (%)	528 (71.2)	34 (81.0)	494 (70.6)	0.149
Discharge medication, *n* (%)				
Dual antiplatelet therapy	778 (97.5)	40 (87.0)	738 (98.1)	0.001
Statins	783 (98.1)	46 (100.0)	737 (98.0)	1.000
β-blockers	561 (70.4)	35 (76.1)	526 (70.0)	0.506
ACEI/ARB	331 (41.5)	24 (52.2)	307 (40.9)	0.131
CCB	176 (22.1)	11 (23.9)	165 (22.0)	0.762

PNI, Prognostic Nutritional Index; GNRI, Geriatric Nutritional Risk Index; BMI, body mass index; HR, heart rate; SBP, systolic blood pressure; Scr, serum creatinine; MI, myocardial infarction; CAD, coronary atherosclerotic heart disease; PCI, percutaneous coronary intervention; Hs-TnT, high-sensitivity troponin T; TG, triglyceride; TC, total cholesterol; LDL-C, low-density lipoprotein-C; HDL-C, high-density lipoprotein-C; LVEF, left ventricular ejection fraction; MVD, multivessel disease; LM, left main disease; LAD, left anterior descending disease; ACEI/ARB, angiotensin-converting enzyme inhibitor/angiotensin receptor blocker; CCB, calcium channel blocker. Data are presented as the mean ± SD, M (IQR) or *n* (%).

**Table 2:** Univariate and multivariate Cox regression analysis for all-cause mortality.

Variables	Univariate Analysis			Multivariate Analysis		
	HR	95% CI	*p* Value	HR	95% CI	*p* Value
Male	1.410	0.769 to 2.587	0.267			
Smoking	0.420	0.208 to 0.846	0.015	0.791	0.375 to 1.672	0.540
GRACE score	1.027	1.017 to 1.037	<0.001	1.017	1.006 to 1.029	0.002
Previous CAD	1.886	1.028 to 3.459	0.041	1.651	0.877 to 3.108	0.120
Atrial fibrillation	4.658	2.308 to 9.401	<0.001	2.249	1.007 to 5.024	0.048
Diabetes	1.790	1.003 to 3.193	0.049	1.437	0.769 to 2.683	0.256
Hypertension	1.501	0.790 to 2.851	0.215			
Uric acid	1.003	1.001 to 1.006	0.007	1.002	1.000 to 1.005	0.054
TG	0.642	0.438 to 0.940	0.023	0.757	0.522 to 1.097	0.142
TC	0.993	0.788 to 1.253	0.956			
LDL-C	1.024	0.753 to 1.394	0.879			
HDL-C	1.107	0.414 to 2.961	0.839			
MVD	1.887	0.927 to 3.840	0.080			
LM or LAD	1.770	0.819 to 3.824	0.146			
Haematocrit	0.921	0.881 to 0.963	<0.001	0.960	0.908 to 1.016	0.159
Dual antiplatelet therapy	0.145	0.062 to 0.343	<0.001	0.493	0.179 to 1.354	0.170
Statins	20.685	0.002 to 262,630.802	0.530			
β-blockers	1.353	0.687 to 2.664	0.382			
ACEI/ARB	1.563	0.876 to 2.788	0.130			
CCB	1.094	0.555 to 2.153	0.796			

CAD, coronary atherosclerotic heart disease; TG, triglyceride; TC, total cholesterol; LDL-C, low-density lipoprotein-C; HDL-C, high-density lipoprotein-C; MVD, multivessel disease; LM, left main disease; LAD, left anterior descending disease; ACEI/ARB, angiotensin-converting enzyme inhibitor/angiotensin receptor blocker; CCB, Calcium channel blockers.

**Table 3:** Cox regression analysis of nutritional indices to predict all-cause mortality.

Variable	HR	95% CI		*p* Value
Serum albumin	0.918	0.844	0.998	0.044
Total lymphocyte count	0.676	0.379	1.206	0.185
Weight loss	0.956	0.447	2.043	0.907
PNI	0.926	0.867	0.989	0.022
GNRI	0.952	0.907	1.001	0.053

PNI, Prognostic Nutritional Index; GNRI, Geriatric Nutritional Risk Index.

**Table 4:** Model performance after the addition of the nutritional indices to the GRACE risk score for predicting all-cause mortality.

Model	All-Cause Mortality					
	C-Index (95% CI)	*p* Value	IDI (95% CI)	*p* Value	NRI (95% CI)	*p* Value
GRACE score	0.722 (0.644, 0.799)	Ref.	Ref.	Ref.	Ref.	Ref.
GRACE score + GNRI	0.736 (0.661, 0.810)	0.198	0.000 (0.000, 0.010)	0.286	0.070 (0.004, 0.187)	<0.001
GRACE score + PNI	0.740 (0.669, 0.812)	0.027	0.006 (0.000, 0.014)	<0.001	0.095 (0.004, 0.147)	<0.001

IDI, integrated discrimination improvement; NRI, net reclassification improvement; PNI, Prognostic Nutritional Index; GNRI, Geriatric Nutritional Risk Index.

## Data Availability

The datasets used and/or analyzed in the study are available from the corresponding author upon reasonable request.
